# Studies of a rice sterile mutant *sstl* from the TRIM collection

**DOI:** 10.1186/s40529-019-0260-3

**Published:** 2019-07-10

**Authors:** Chia-Ling Chang, Jerry C. Serapion, Han-Hui Hung, Yan-Cheng Lin, Yuan-Ching Tsai, Wann-Neng Jane, Men-Chi Chang, Ming-Hsin Lai, Yue-ie C. Hsing

**Affiliations:** 1 Department of Agronomy, National Taiwan University, Taipei, 106 Taiwan; 2 Institute of Plant and Microbial Biology, Academia Sinica, Nankang, Taipei, 115 Taiwan; 3 Crop Science Division, Taiwan Agricultural Research Institute, Taichung, 413 Taiwan; 4 Department of Life Science, National Taiwan University, Taipei, 106 Taiwan; 5 Department of Agronomy, National Chiayi University, Chiayi, 600 Taiwan

**Keywords:** Anther development, Microspores, Transcriptomic analysis, Semi-sterile, Sterility

## Abstract

**Background:**

Rice (*Oryza sativa*) is one of the main crops in the world, and more than 3.9 billion people will consume rice by 2025. Sterility significantly affects rice production and leads to yield defects. The undeveloped anthers or abnormal pollen represent serious defects in rice male sterility. Therefore, understanding the mechanism of male sterility is an important task. Here, we investigated a rice sterile mutant according to its developmental morphology and transcriptional profiles.

**Results:**

An untagged T-DNA insertional mutant showed defective pollen and abnormal anthers as compared with its semi-sterile mutant (*sstl*) progeny segregates. Transcriptomic analysis of sterile *sstl*-*s* revealed several biosynthesis pathways, such as downregulated cell wall, lipids, secondary metabolism, and starch synthesis. This downregulation is consistent with the morphological characterization of *sstl*-*s* anthers with irregular exine, absence of intine, no starch accumulation in pollen grains and no accumulated flavonoids in anthers. Moreover, defective microsporangia development led to abnormal anther locule and aborted microspores. The downregulated lipids, starch, and cell wall synthesis-related genes resulted in loss of fertility.

**Conclusions:**

We illustrate the importance of microsporangia in the development of anthers and functional microspores. Abnormal development of pollen grains, pollen wall, anther locule, etc. result in severe yield reduction.

**Electronic supplementary material:**

The online version of this article (10.1186/s40529-019-0260-3) contains supplementary material, which is available to authorized users.

## Background

Rice (*Oryza sativa*) is one of the most important staple crops in the world and is also an economical and cultivated food in Asia. One of the major limitations in rice yield production is sterility. Several studies provided strong evidence that male sterility was a serious defect in rice production and led to significantly decreased rice production (Thangasamy et al. 2011; Wang et al. 2013; Guo et al. 2013; Pu et al. 2017) or even almost complete loss (Liu et al. 2010; Ko et al. 2014; Yang et al. 2014; Li et al. 2016).

Male sterility is associated with a series of anther development steps and/or pollen maturation at the rice reproductive stage. Studies of the developmental morphology of pollen revealed several important steps, such as cell differentiation into microspores and circular anther locules, the formation of four specialized cell layers in the anther, meiosis of the pollen mother cell, microspores with a well-developed pollen wall, microspore mitosis, and intine establishment of mature pollen (Raghavan 1988; Zhang et al. 2011; Walbot and Egger 2016). Previous studies concluded that the process of pollen maturation in rice was initiated in the L1, L2 and L3 layers of the stamen primordia, and central archesporial (AR) cells were generated from the mitotic division in the L2 layer to develop the primary parietal cells (PPCs) and primary sporogenous cells (PSCs) (Zhang et al. 2011; Kelliher et al. 2014; Yang et al. 2018). After AR cells divide and differentiate to PSCs and PPCs, PSCs form as sporogenous cells and later generate microspores; PPCs divide and develop into secondary parietal layers (SPCs). The outer layer of SPCs becomes the endothecium and middle layer, and the inner layer becomes the tapetal cell (Zhang and Wilson 2009).

Receptor protein kinase (RPK) is important in regulating tapetal cells and microsporocytes during sporophytic generation. In *Arabidopsis*, a putative leucine-rich repeat receptor protein kinase (LRR-RPK) gene, *EXCESS MICROSPOROCYTES1* (*EMS1*)*/EXTRA SPOROGENOUS CELLS* (*EXS*), participates in early division of the anther and microsporocyte cells to control tapetum and microsporocyte development (Cai and Zhang 2018; Verma 2019). TAPETUM DETERMINANT1 (TPD1) was found as a cysteine-rich protein ligand of EMS1. TPD1 interacts with the EMS1 LRR domain to generate precursors of tapetal cells by promoting cell division of inner SPCs; the developmental tapetum layer determines the number of sporogenous cells by suppressing proliferation (Huang et al. 2016). A rice protein binding cassette with the LRR-RLK protein MULTIPLE SPOROCYTE1 and its ligand TPD1-like protein (OsTDL1A) is involved in PPC division to contribute SPCs for the middle layer and establish the tapetum. The anther with double mutated *OSTDL1A* and *MULTIPLE SPOROCYTE1* genes illustrated a lack of middle layers and tapetum and increased the number of microsporocytes in the early development of pollen (Yang et al. 2016). On mitosis, primary sporogenous cells divide into secondary sporogenous cells and become pollen mother cells (PMCs). PMCs pass through meiosis to generate microspores. At the same time, programmed cell death (PCD) of the tapetum layer occurs during meiosis. Several regulators respond to microspore generation, and PCD of tapetal cells such as a rice basic helix-loop-helix (bHLH) protein, TAPETUM DEGENERATION RETARDATION (TDR), controls tapetum degeneration to contribute to microspore development (Li et al. 2006). Gibberellic acid-inducible transcription factor *GAMYB* upregulates *TDR1* expression, and a frame shift with a C deletion of the allele *gamyb*-*4* did not go through normal meiosis or PCD of tapetum in rice (Liu et al. 2010). The bHLH142 that conjugates with TDR1 turned on the downstream gene *ETERNAL TAPETUM1 (EAT1)* to organize microspore development. With T-DNA insertion into *bHLH142*, the tapetal PCD and microspore development were defective in anthers of rice loss-of-function *bhlh142* (Ko et al. 2014).

Microspores are produced after PMC meiosis, and young microspores are released from the tetrads later. The rice *Wax*-*deficient anther1* (*Wda1*) defective mutant showed a significant defect of very-long-chain fatty acids in the anther epidermis and pollen wall that led to a severe absence of pollen (Jung et al. 2006). Thus, the fatty acid of pollen wall conformation plays an important role in microspore maturation.

The pollen wall is composed of the outer pollen wall exine and inner wall intine. Transmission electron microscope (TEM) observation revealed that exine consists of the tectum, bacula, and nexine together as a bridge-like structure of the pollen outer wall (Shi et al. 2015). The pollen walls of microspores are constructed during tapetum layer degradation and again indicate the importance of lipid metabolism (Li et al. 2011). Transcriptomic analysis revealed that transcripts encoding cytochrome P450, acyltransferases, and lipid transfer proteins are produced from the tapetum secretion stage to support fatty acid accumulation and transportation to build exine (Huang et al. 2009). Without PCD, tapetum did not degenerate to generate fatty acid components such as sporopollenin for exine formation. Rice *PERSISTENT TAPETAL CELL1* (*PTC1*), which affects fatty acid synthesis in the exine structure, promotes tapetum degradation to generate sporopollenin for pollen wall development (Li et al. 2011). Scanning electron microscopy (SEM) revealed that the tapetal cell also produces orbicules during tapetum degeneration, which surround the surface of the tapetum layer, and orbicules provided the fatty acid product for assembly of sporopollenin for exine (Daku et al. 2016; Ruggiero and Bedini 2018). A rice lipid transfer protein OsC6 involved in lipid transfer from tapetum to exine is regulated by *bHLH*, *TDR*, and *GAMYB.* The truncated *osc6* mutation resulted in defective orbicules and disordered exine structure in the pollen wall (Zhang et al. 2010). Thus, a well-developed exine is an indicator of maturation of microspore fertility during anther development.

The biosynthesis of sporopollenin is modified by several enzymes such as *in*-*chain* fatty acid hydroxylase (AtCYP703A2; OsCYP703A3), fatty acid ω-hydroxylase (AtCYP704B1; OsCYP704B2), polyketide synthase (OsPKS1), tetraketide reductase (OsTKPR1), fatty acid anther-specific acyl-CoA synthetase (ACOS; OsACOS12), and reductase (OsDPW) in fatty acid metabolism (Li et al. 2010, 2016; Choi et al. 2011; Wang et al. 2013; Yang et al. 2014; Daku et al. 2016). These enzymes participate in a series of steps to convert fatty acid acyl-CoA to the final sporopollenin product, which is then transported by ABC transporters such as ABCG26/WBC27, OsABCG3 and lipid transfer protein OsC6 from the tapetum to exine for pollen wall utilization (Zhang et al. 2010; Choi et al. 2011; Chang et al. 2018). The intine of the pollen wall consists of carbohydrate polysaccharides, which are converted from UDP-glucose (Shi et al. 2015). The intine formation of rice is controlled by Golgi-localized GLYCOSYLTRANSFERASE1 (OsGT1), which transfers UDP-glucose for polysaccharide composition requirement, and also regulates starch and protein synthesis in pollen grain maturation to determine rice fertility (Moon et al. 2013). The polysaccharides of intine also regulate water content in pollen dehydration and disperse pressure when mature pollen spores expand with internal content accumulation (Xu et al. 2016).

Taiwan Rice Insertional Mutagenesis (TRIM) was initiated in 2002. In this mutant population, 93,000 rice mutated lines were created by T-DNA insertion by using a japonica variety, Tainung 67 (Wu et al. 2017). The reverse genetics approach provided a large-scale population of T-DNA mutagenesis screening for comparing phenotypic characterization and rice genomic function. The TRIM population was used in two studies on male-sterile mutants. T-DNA insertion into *bHLH142* led to no response to PCD of tapetum, and this gene was regulated by the early tapetum developing-related genes *UNDEVELOPED TAPETUM1 (UDT1)* and *GAMYB*, then was responsible for downstream *TDR1* and *EAT1* regulation (Ko et al. 2014). Another TRIM mutant study indicated that a rice SUMO E3 ligase *SIZ1* participated in controlling endothecium development during anther dehiscence to manipulate the fertility of rice. Mutant *siz1* showed the inability of anther dehiscence and no cavity of two adjacent locules; thus, it released no pollen grains during anthesis. In addition, this mutant had a shorter plant with few tillers and low seed set rate (Thangasamy et al. 2011).

Here we report a morphological characterization of a male sterile mutant from the TRIM collection and investigate the mechanism of sterility. We illustrate the importance of microspore fertility in anther development and the functional biosynthesis of microspores. We also performed a comparative transcriptomic analysis between fertile and sterile anthers to prove that fatty acid biosynthesis pathways participate in the morphology of pollen wall formation.

## Methods

### Plant materials and growth condition

T-DNA insertional mutant line M0037841 with 30 T_2_ seeds were obtained from the TRIM collection and grown at the genetically modified plant isolation field (GM field) in the Taiwan Agricultural Research Institute (TARI) for agronomic trait evaluation. Leaf samples from individual plants of the T_2_ population were harvested and frozen under liquid nitrogen for genomic DNA extraction experiments. Progeny from the semi-sterile line of M0037841 were both grown in the TARI GM qualified field or a growth chamber under day 28 °C/night 25 °C, 16 h of light and 80% humidity at Academia Sinica, Taipei. Yield component analysis including panicle number, grain number, fertility rate, and grain weight was performed by the semi-sterile segregates in the TARI GM field. The anther samples for floral morphology observation, pollen viability assay, gene expression analysis, phenotypic characterization of cross-sections, TEM and SEM were collected from plants grown in the growth chamber.

### Genomic DNA extraction and PCR-based genotyping

Genomic DNA was extracted from leaves by using the Wizard Genomic DNA purification kit (Promega Corporation, Madison, WI, USA). All T-DNA mutagenesis plants from T_2_ were identified by T-DNA tagging regions, a T-DNA construct pTAG8, such as hygromycin phosphotransferase gene (*HPT*), *GUS* gene, and *CaMV35S* enhancer with PCR-based genotyping assay. The PCR reaction in 20 μL volume involved 50 ng genomic DNA, 0.2 μm primers and Taq DNA Polymerase 2× Master Mix RED (Ampliqon, Herlev, Denmark) for amplification. The sequences of primers are in Additional file 1: Table S1.

### Pollen viability and germination analysis

Mature pollen grains were collected before anthesis in the fertile, semi-sterile and sterile types from semi-sterile progeny segregates and stained with iodine–potassium iodide (I_2_–KI) solution for pollen viability. Pollen viability was evaluated by using pollen shape and staining for classification into four grades as dark black (+++), partial black (++), orange (+) and yellow (−). More than 2000 individual pollen grains were counted under a Zeiss AxioImager Z1 microscope. More than 1000 pollen grains per sample were co-cultured with 15% sucrose (Sigma-Aldrich, St. Louis, MO, USA), 5% starch (Sigma-Aldrich, St. Louis, MO, USA), and 0.005% orthoboric acid (Merck, Darmstadt, Germany) at 28 °C for 30 min for detecting pollen tube germination rate.

### Morphological analysis of anther characterization

Several reproductive growth parameters of development were revealed by kernel size, spikelet length, spikelet width, anther color, and anther length for 5 anther stages, young microspores stage (YM), vacuolated pollen stage (VP), early pollen mitosis stage (EpM), late pollen mitosis stage (LpM), and mature pollen stage (Mp) (Zhang and Wilson 2009; Huang et al. 2009). These five stages were equal to the anther stages from Zhang and Wilson (2009) reported in “stage 9”, “stage 10”, the first mitotic division of microspore in “stage 11”, the second mitosis of microspore in “stage 12”, and the spherical mature pollen grains in “stage 13”. Anther samples of all developmental stages were fixed in 2.5% glutaraldehyde, 4% paraformaldehyde, and 0.1 M sodium K-phosphate buffer (pH 7.0) at 4 °C for 24 h. Then, after three rinses with 0.1 M K-phosphate buffer (pH 7.0) for 20 min each, samples were transferred to 1% OsO_4_ in 0.1 M K-phosphate (pH 7.0) for 4 h at room temperature. The samples were washed again with 0.1 M K-phosphate buffer (pH 7.0) as for the previous rinsed steps. Samples were dehydrated with an ethanol series and propylene oxide, and embedded in Spurr resin. The cross sections were prepared in 1-μm sections and stained with toluidine blue for anther transverse section detection and anther locule area investigation under a Zeiss AxioImager Z1 microscope. The ultrathin sections in 70–90 nm were stained with uranyl acetate and lead citrate for TEM imaging by using a Philips CM 100 TEM system at 80 kV. For cryo-SEM, anthers and pollen grains at Mp stage were frozen in liquid nitrogen and placed into the sample preparation chamber at − 160 °C. After the temperature reached − 85 °C and sublimation/etching proceeded for 15 min, samples were coated with platinum particles at − 130 °C. Samples were moved to a cryo-stage SEM chamber and examined by using a Cryo-SEM system at temperature − 190 °C (Quanta 200 SEM/cryo system Quorum PP2000TR FEI).

### RNA extraction and transcriptomic analysis

Anther samples were collected from YM or LpM stages for total RNA isolation by using TRIzol reagent (Thermo Fisher Scientific, Wilmington, DE, USA), and 5 μg total RNA was prepared for RNA sequencing. For RNA sequencing, single-end libraries were created and sequenced on an Illumina HiSeq 2000 system in 90-bp read length for 10 million reads per sample. Sequence data were deposited in the NCBI gene expression omnibus (accession no. GSE129579). RNA sequencing data were evaluated by RNA-seq analysis, differential expression of genes and heat map drawing with the CLC Genomics Workbench 11 (CLC Bio, QIAGEN Bioinformatics, Aarhus, Denmark). The RNA-seq transcripts were annotated by using the RAP-DB database (https://rapdb.dna.affrc.go.jp/index.html) and compared with the Rice Expression Profile Database (http://ricexpro.dna.affrc.go.jp/). Anther-specific transcriptomic genes were also confirmed by Rice Anther Expression Plots (https://www.cpib.ac.uk/anther/riceindex.html). EXPath Tool (http://expathtool.itps.ncku.edu.tw/), Kyoto Encyclopedia of Genes and Genomes (KEGG) (http://www.genome.jp/kegg/), and MapMen (https://mapman.gabipd.org/) were used for gene function prediction and analysis.

### Gene expression analysis

Total RNA from YM and LpM were converted to cDNA with use of oligo (dT) primers by the SuperScript III First-Strand Synthesis System (Thermo Fisher Scientific, Wilmington, DE, USA). Real-time quantitative RT-PCR was performed with the SYBR Green-based quantitative PCR method by mixing 20 ng cDNA, 0.25 μm primers and fluorescence Power SYBR Green PCR Master Mix (Applied Biosystems, Foster City, CA, USA) for gene expression detection. The gene expression was calculated by the 2^−ΔΔCt^ method with *OsUBI*, *OsACT11*, *OsUBQ5*, and *eEF*-*1α* used as internal control genes. All primer sequences are in Additional file 1: Table S1.

## Results

### A semi-sterile mutant from TRIM collection

Sterility is one of the important regulators of the reproductive stage in rice yield production. To study this phenomenon of sterility, we selected the T-DNA insertional mutant line M0037841 from the TRIM collection. Thirty T_2_ seeds of M0037841 were grown in a GM field in TARI. The T_2_ population in M0037841 was identified without co-segregation between the phenotype of sterility and the T-DNA insertional event of the genome (Additional file 2: Table S2).

**Fig. 1:**
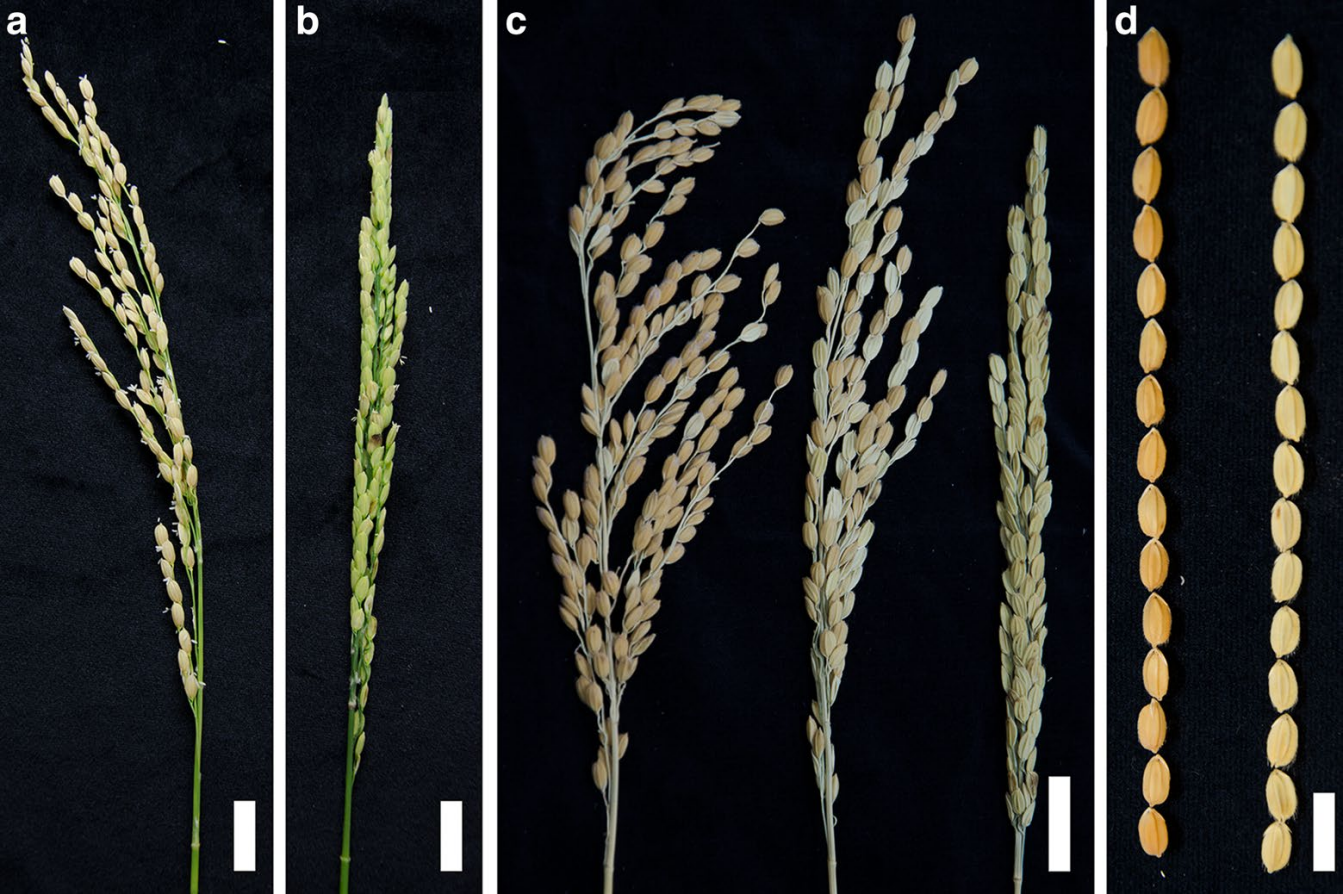
Phenotypic characterization of segregants of the semi-sterile mutant (*sstl*). **a** Plants with fertile panicles; **b** completely sterile panicles; **c** three types of fertility in the *sstl* progeny segregates: from left to right as fertile, semi-sterile, and sterile. Bar = 2 cm. **d** Seed size between fertile and sterile indicated. Bar = 1 cm

From the developmental morphology of the T_2_ population in M0037841, we noticed two plants were semi-sterile and thus designated them semi-sterile (*sstl*) for the rest of the study (Additional file 2: Table S2). From the progeny segregates of the mutant *sstl*, characteristic distributions of fertility were classified as fertile panicle plants (*SSTL*-*F*), semi-sterile panicle plants (*sstl*-*ss*), and sterile panicle plants (*sstl*-*s*) (Fig. 1a–c). In *sstl* progeny segregates, *SSTL*-*F* and *sstl*-*s* lines did not differ in plant height or tiller number during the vegetative stage (Additional file 3: Figure S1). In the reproductive stage, yield component traits were analyzed as fertility rate, number of panicles, grain number and grain weight in *SSTL*-*F*, *sstl*-*ss*, and *sstl*-*s*. The fertility rate of *sstl*-*ss* was 49.2% (Table 1), showing half fertile seeds per panicle (Fig. 1c). *SSTL*-*F*, *sstl*-*ss*, and *sstl*-*s* all had 15 panicles per plant, but the grain number of each panicle gradually increased from *SSTL*-*F* and *sstl*-*ss* to *sstl*-*s*. The grain size from fertile and sterile spikelets did not differ. The kernel shape, along with palea and lemma, did not differ (Fig. 1d).

**Table 1:** Yield component analysis of *sstl* progeny segregates

Phenotypes	AFRP (%)	ANP	ANGP	AGWP (g)
*SSTL*-*F*	92.80 a	15.71 a	148.43 ab	4.01 a
*sstl*-*ss*	49.20 b	15.43 a	163.67 b	2.57 b
*sstl*-*s*	0.07 c	15.14 a	179.71 bc	0.96 c

Yield component analysis of three types in *sstl* progeny segregates*. F* fertile, *ss* semi-sterile, *s* sterile, *AFRP* average fertility rate per panicle, *ANP* average number of panicles per plant, *ANGP* average number of grains per panicle, *AGWP* average grain weight per panicle. The statistical analysis was performed by one-way ANOVA with Tukey test post hoc analysis. Data were collected from 7 individual plants in each type and presented as mean ± SEM

Values followed by different letters indicated a significant difference for each variable (P ≤ 0.05)

Thus, the sterile characterization of *sstl*-*s* was not caused by a T-DNA insertion, and *sstl*-*s* still inherited the sterility trait from the progenitor semi-sterile *sstl.*

### Sterility from *sstl*-*s* pollen viability

**Fig. 2:**
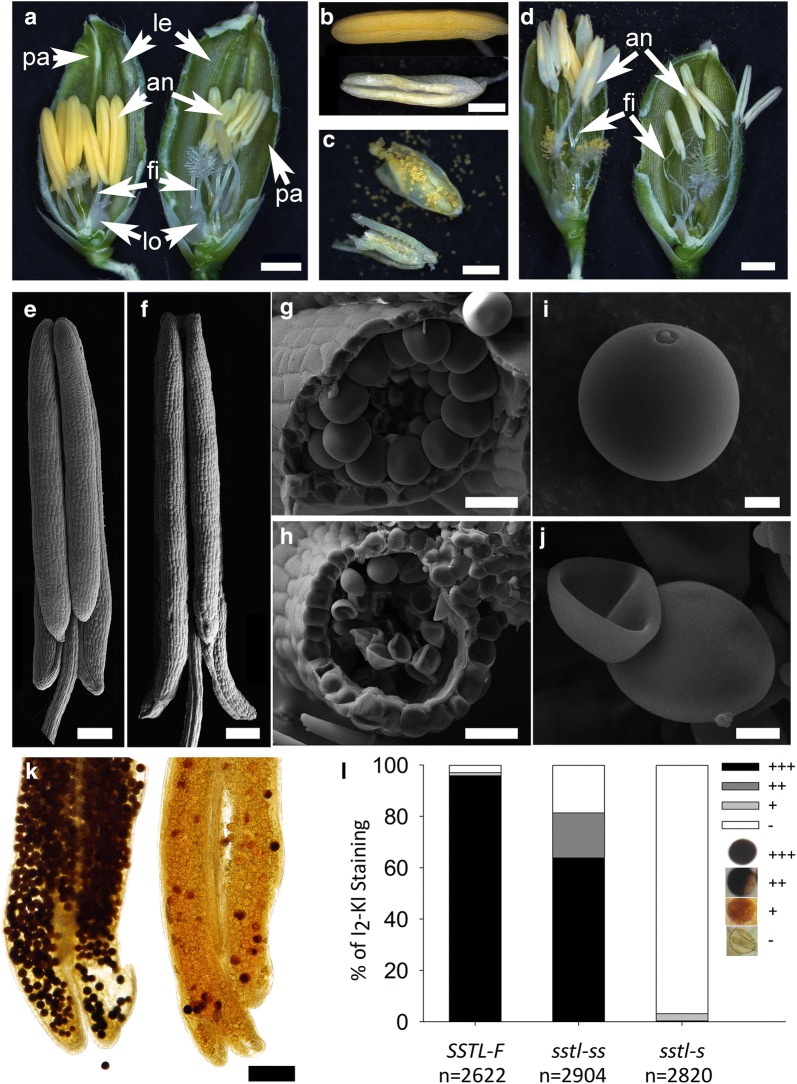
Floral morphology of fertile and sterile plants from *sstl* offspring. **a** The floral structure between *SSTL*-*F* (left) and *sstl*-*s* (right) at heading stage. Bar = 1 mm. **b** The exterior of anther in *SSTL*-*F* (above) and *sstl*-*s* (below). Bar = 0.4 mm. **c** Mature pollen was presented after chopping the anthers of *SSTL*-*F* (above) and *sstl*-*s* (below). Bar = 0.4 mm. **d** Filament elongation of *SSTL*-*F* (left) and *sstl*-*s* (right) during anthesis. Bar = 1 mm. **e**, **g**, **i** SEM observation of *SSTL*-*F* and **f**, **h**, **j** *sstl*-*s* in anther and pollen. **e**, **f** Bar = 0.2 mm; **g**–**j** bar = 50 μm. **k** I_2_KI staining of *SSTL*-*F* (left) and *sstl*-*s* (right). Bar = 200 μm. **l** Pollen viability in different maturity types of *SSTL*-*F*, *sstl*-*ss,* and *sstl*-*s*. *an* anther, *fi* filament, *le* lemma, *lo* lodicule, *pa* palea

To understand the sterility of rice mutant *sstl*-*s*, we investigated the floral morphology of *SSTL*-*F* and *sstl*-*s.* During pollen maturation, the flower of *sstl*-*s* exhibited a white anther, indicating lack of flavonoid components (Fig. 2a, b). Mature pollen grains released from anthers of *SSTL*-*F* and (above) and *sstl*-*s* (below) showed a white color in *sstl*-*s* (Fig. 2c). Pollen grains from *sstl*-*s* were so tightly stuck to anther locules that they may not be exposed easily as mature pollen in *SSTL*-*F* after cutting the anthers (Fig. 2c). During anthesis in *SSTL*-*F*, the mature pollen grains were released from anther dehiscence and the filaments elongated. However, only elongated filaments were found in *sstl*-*s* (Fig. 2d). The anther locules of *sstl*-*s* were juicy and sticky and thus the pollen grains were barely released after filament elongation. This finding was consistent with the anther locules of *sstl*-*s* after chopping (Fig. 2c, d). By SEM observations of *SSTL*-*F* and *sstl*-*s* anthers, we confirmed that *sstl*-*s* had a shrinking anther and smaller pollen sac as compared with *SSTL*-*F* (Fig. 2e–h). Additionally, the pollen grains of *sstl*-*s* did not fill up with content and formed a stomatocyte-like shape during maturation (Fig. 2g–j). The pollen sacs of *sstl*-*s* had no surrounding pollen grains on anther locules because pollen grains of *sstl*-*s* did not form as spherical as compared with mature pollen of *SSTL*-*F* (Fig. 2g, h).

Pollen viability and pollen tube germination analyses were performed to determine the severity of defective pollen in *sstl*-*s*. Because mature pollen grains contained abundant starch to provide energy for supporting pollen maturation and pollen tube germination (Lee et al. 2016), we studied pollen viability by using I_2_–KI staining. Figure 2k showed a significant difference between mature pollen grains of *SSTL*-*F* (left) and *sstl*-*s* (right) in pollen viability, as indicated by staining color and pollen spherical shape. The starch accumulation of these pollen grains was illustrated as dark black (+++), partial black (++), orange (+) and yellow (−) after I_2_–KI staining (Fig. 2l). The proportion of dark-black mature pollen in *SSTL*-*F, sstl*-*ss*, and *sstl*-*s* was 96%, 64%, and 0.35%, respectively, revealing low pollen viability in the sterile mutant (Fig. 2l). Besides, *sstl*-*ss* showed 18% partial black pollen, with none in *SSTL*-*F* and *sstl*-*s.* The proportion of dark-black pollen in semi-sterile *sstl*-*ss* was about 60% and agreed well with half fertile seeds per panicle in *sstl*-*ss* (Table 1 and Fig. 2l). In addition, examination of pollen-tube germination of fertile and sterile pollen grains confirmed an 81.7% germination rate in *SSTL*-*F* but only 0.26% in *sstl*-*s* (data not shown).

The defects in *sstl*-*s* pollen reveal that its anther locules did not undergo anther dehiscence because the pollen sacs did not expand and pollen grains did not form a spherical shape. Almost zero percentage of pollen viability and pollen germination rates were consistent with the sterility of the mutant *sstl*-*s* as well as pollen-defective morphology.

### Anther morphology of *sstl*-*s*

**Fig. 3:**
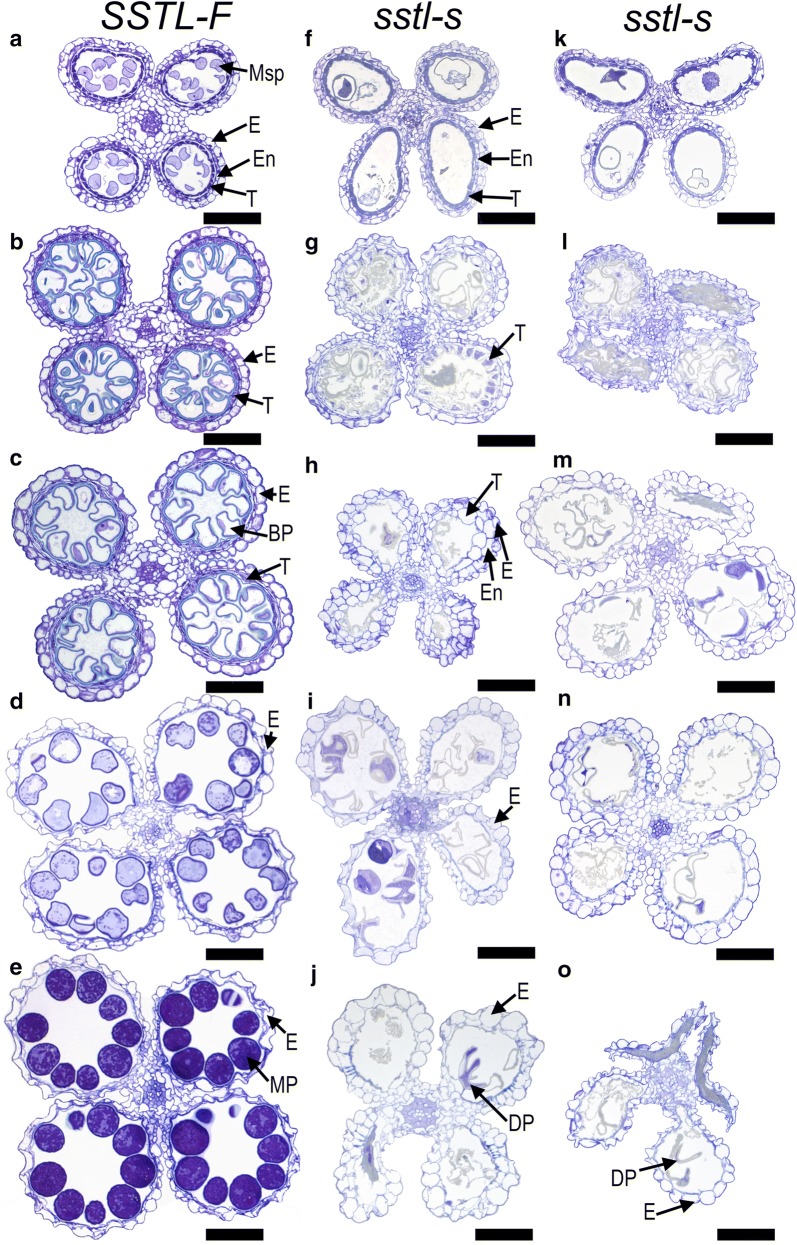
Cytological observation of cross sections from developing anthers. **a** Anthers at young microspore stage (YM) with epidermis, endothecium, middle layer, and tapetum layers of *SSTL*-*F* and **f**, **k** *sstl*-*s*. **b** At vacuolated pollen stage (VP), when the microspores formed vacuolated in *SSTL*-*F* and **g**, **l** *sstl*-*s*. **c** At early pollen mitosis stage (EpM), when microspores went through the first mitosis and became bicellular pollen in *SSTL*-*F* and **h**, **m** *sstl*-*s*. **d** At late pollen mitosis stage (LpM), when microspores went through the second mitotic division for two sperm cells in *SSTL*-*F* and **i**, **n** *sstl*-*s*. **e** At mature pollen stage (Mp), when microspores were in the plump roundness shape of *SSTL*-*F* and **j**, **o** *sstl*-*s*. *BP* bicellular pollen, *DP* defective pollen, *E* epidermis, *En* endothecium, *MP* mature pollen, *Msp* microspore parietal cell, *T* tapetum. Bars = 80 μm

Cross sections of anthers in *SSTL*-*F* and *sstl*-*s* at specific anther development stage provided tools to understand male sterility. At YM stage, both *SSTL*-*F* and *sstl*-*s* had four cell layers (i.e., epidermis, endothecium, middle layer, and tapetum) (Fig. 3a, f, k). Microspores were released from tetrads by callose degradation in *SSTL*-*F* (Fig. 3a) but few from *sstl*-*s* anthers (Fig. 3f, k). The tapetum layer started degenerating and the microspores formed vacuolated as an oval shape in *SSTL*-*F* anthers (Fig. 3b). Tapetum layers of sterile *sstl*-*s* still degraded (Fig. 3g, l), similar to the fertile line *SSTL*-*F* anther at VP stage. Later on, microspores went through the first mitosis and became bicellular pollen. The microspores were smaller from the EpM than VP stage and the starch began to accumulate in fertile microspores (Fig. 3c). Meanwhile, the tapetal cells were almost completely degenerated and left only a thin layer on the internal surface of the anther locule in *SSTL*-*F* (Fig. 3c). In contrast, the *sstl*-*s* anther showed a delay of tapetum degradation (Fig. 3h, m) and a larger size of tapetal cell at the EpM stage. In the LpM stage, microspores differentiated into two sperm cells by the second mitosis division. *SSTL*-*F* microspores contained a high amount of starch and lipid accumulation (Fig. 3d). The endothecium and epidermal cells of *SSTL*-*F* degenerated in the LpM stage (Fig. 3d), but *sstl*-*s* anthers contained defective pollen grains, and anther locules maintained a larger epidermis and endothecium cell as compared with fertile ones (Fig. 3i, n). The tapetum layer of *SSTL*-*F* degenerated and disappeared at this stage (Fig. 3d) but remained partially in the *sstl*-*s* anther because of delayed degradation (Fig. 3n). The final stage of microspore maturation was the formation of plump round mature pollen in *SSTL*-*F* (Fig. 3e), but only defective pollen was produced in *sstl*-*s* anthers (Fig. 3j, o). The epidermis degenerated and became smaller than those from previous stages in the *SSTL*-*F* anther. However, as compared with *sstl*-*s* anthers, the epidermal cells of *sstl*-*s* were three times larger than *SSTL*-*F* cells (Fig. 3j, o). The mutant *sstl*-*s* contained defective pollen and its anthers also contained almost no microspores in the developmental process. Microspores were barely generated in *sstl*-*s* anthers, and *sstl*-*s* showed undeveloped anther locules without PMCs in early-stage cross sections (Additional file 4: Figure S2). This abnormal anther locule illustrated that these anthers had no specialized four layers (i.e., epidermis, endothecium, middle layer, and tapetum) to form a circular anther locule. The functional PMCs were not produced to generate microspores in sterile *sstl*-*s* (Additional file 4: Figure S2). From TEM observations, only epidermal cells remained in these slender twig-like undeveloped anther locules (Additional file 4: Figure S2f, i).

**Fig. 4:**
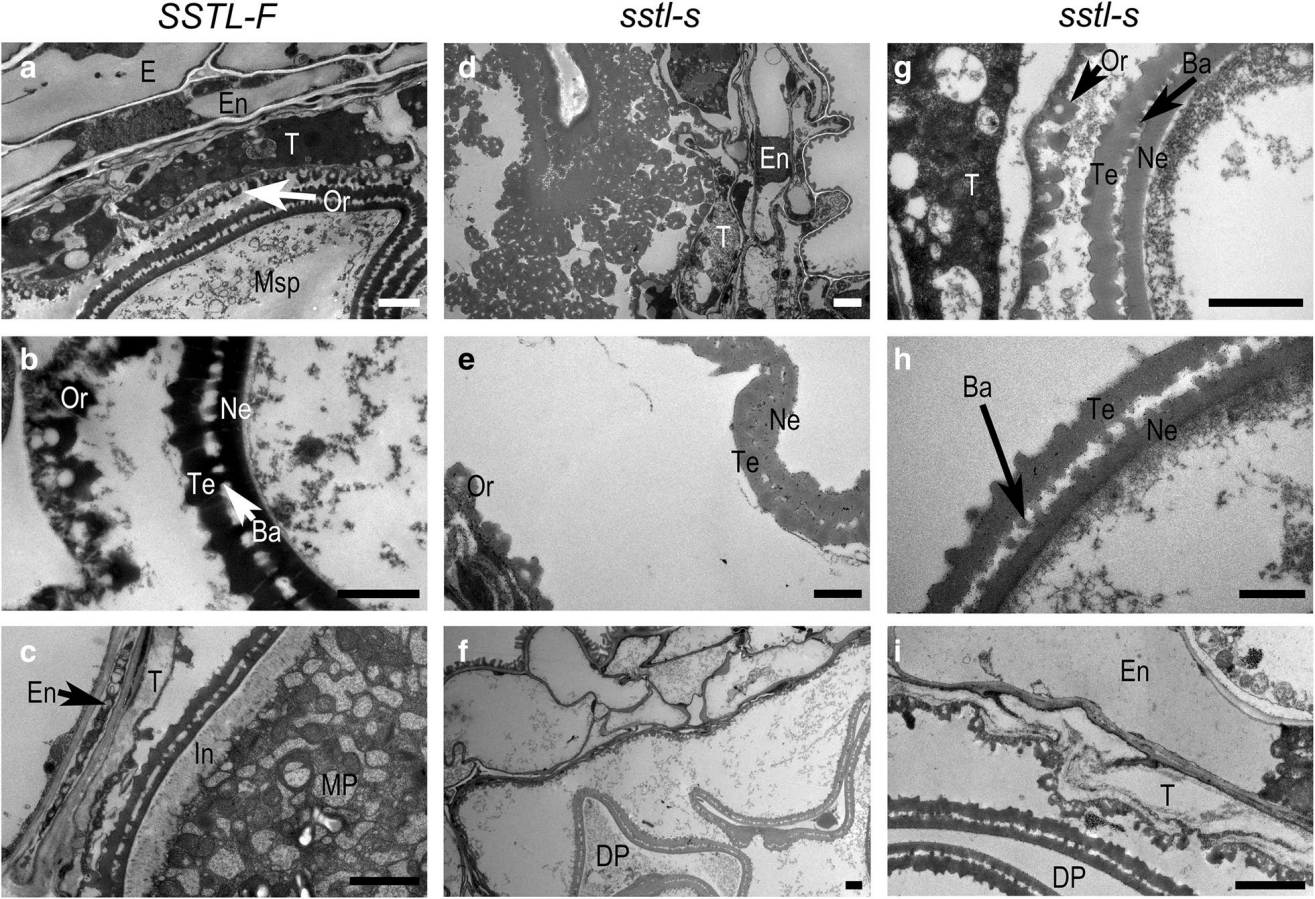
TEM analysis of anther development in *SSTL*-*F* and *sstl*-*s*. **a** At vacuolated pollen stage (VP), when the tapetal cell was decomposed to generate orbicules in *SSTL*-*F* and **d**, **g** *sstl*-*s*. Bars = 2 μm. **b** At late pollen mitosis stage (LpM), when the tapetum was degraded, and the pollen wall of microspores integrated completely in *SSTL*-*F* and **e**, **h** *sstl*-*s*. Bars = 1 μm. **c** At mature pollen stage (Mp), when endothecium degenerated and microspores were in the plump round shape with puffy intine of *SSTL*-*F* and **f**, **i** *sstl*-*s*. Bars = 2 μm. *Ba* Bacular, *DP* defective pollen, *E* epidermis, *En* endothecium, *In* intine, *MP* mature pollen, *Msp* microspore, *Ne* nexine, *Or* orbicule, *T* tapetum, *Te* tectum

To better understand the defective pollen of sterile *sstl*-*s* during pollen development, we used TEM analysis for the structure of anthers and microspores in the wild type and mutant. During VP stage, consistent with the previous studies, both tapetal cells of fertile *SSTL*-*F* and sterile *sstl*-*s* degenerated and thus promoted orbicule formation (Fig. 4a, d, g). The orbicules were constructed as a cone shape with the high electronic denseness of the tapetum inner side layer to provide a lipid component for the pollen exine (Zhang et al. 2010). The orbicule and pollen exine (at tectum, bacular and nexine) were darker under TEM, which indicates concentrated lipid accumulation. The orbicule and exine were much darker in fertile *SSTL*-*F* than sterile *sstl*-*s*, which suggests less lipid content in the *sstl*-*s* anther (Fig. 4b, e, h). At LpM stage, the structure of exine was complete, shown as bridge-like, with ordered tectum, bacular and nexine (Fig. 4b), whereas the exine of *sstl*-*s* contained no space between the tectum and nexine, so it became a messy aggregation in exine (Fig. 4e). The other type of abnormal exine in sterile *sstl*-*s* was the defective bridge-like shape in the pollen wall (Fig. 4h). Mature pollen of fertile *SSTL*-*F* contained conspicuous intine (Fig. 4c), whereas intine was absent in *sstl*-*s* (Fig. 4i). Intine contains a high amount of polysaccharides (Caffall and Mohnen 2009). In addition, intine formation is controlled by glycosyltransferase, and complete intine would determine the accumulation of starch, protein, and other important contents for mature pollen grain (Moon et al. 2013). Sterile *sstl*-*s* exhibited completely defective intine, leading to defective pollen grain accumulation at Mp stage (Fig. 4f, i). Besides, the endothecium and tapetum layers of fertile *SSTL*-*F* decreased entirely to a thin surface at Mp stage (Fig. 4c) rather than the slight reduction in *sstl*-*s* anthers (Fig. 4i).

**Fig. 5:**
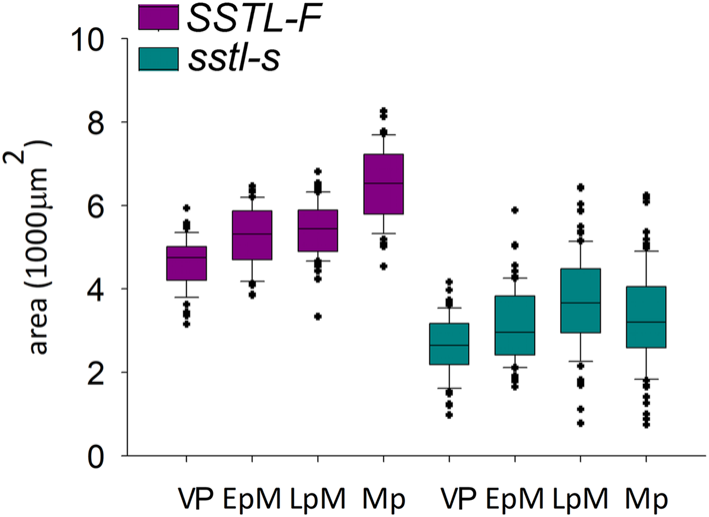
Area of anther locules in *SSTL*-*F* and *sstl*-*s*. More than 20 individual anthers were calculated from each sample stage. *VP* vacuolated pollen stage, *EpM* early pollen mitosis stage, *LpM* late pollen mitosis stage, *Mp* mature pollen stage. Horizontal line is median, box edges are Q1–Q3 and whiskers are range

We measured the area of anther locule with more than 20 individual anthers for *SSTL*-*F* and *sstl*-*s*. The defective anther locules in *sstl*-*s* were less than half of those in *SSTL*-*F* (Fig. 5). The minimum anther locule area of *sstl*-*s* was about 0.5 × 1000 μm^2^ at LpM and Mp stages and was consistent with the cross section of *sstl*-*s* anthers (Fig. 3j, o). The statistical analysis of the anther locule area from *sstl*-*s* also gave similar findings of a small anther locule or unexpanded anther in *sstl*-*s* (Fig. 2e–h). This information suggests that the abnormal anther locule results in a shrunken and un-developmental anther in *sstl*-*s*.

### Transcriptomics analysis of the *sstl*-*s* mutant

**Fig. 6:**
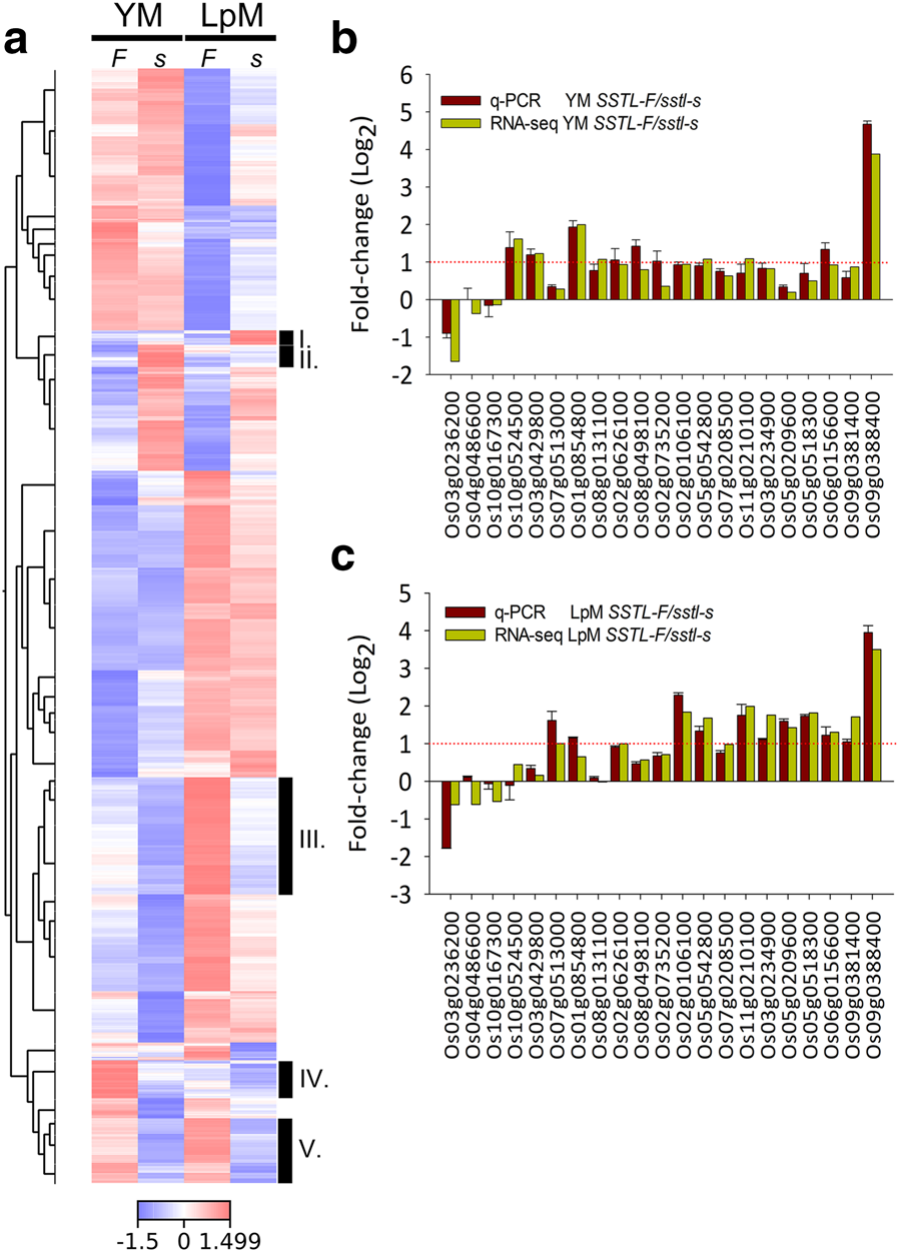
Gene expression pattern in different anther stages. **a** Heat-map analysis of RNA-seq data in YM and LpM stage for fertile and sterile anthers. **b** Relative contribution of fold changes between RNA-seq and qPCR data in YM and **c** LpM. Gene expression of q-PCR data normalized to that of *OsUBI*, *OsACT11*, *OsUBQ5*, and *eEF*-*1α*. *YM* young microspores stage, *LpM* late pollen mitosis stage, *I* a cluster with specific upregulated genes of *sstl*-*s* in LpM, *II* a cluster with specific upregulated genes of *sstl*-*s* in YM, *III* a cluster with specific upregulated genes of *SSTL*-*F* in LpM, *IV* a cluster with specific upregulated genes of *SSTL*-*F* in YM, *V* a cluster of *SSTL*-*F* upregulated genes in both YM and LpM stages, *F* fertile, *s* sterile. Data are mean ± SD

With the morphologic observations of the *sstl*-*s* mutant, we considered that processes such as starch accumulation, sporopollenin, and pollen wall structure as well as biosyntheses such as sucrose, secondary production, and lipid component metabolism must be involved in the pollen maturation process. To discuss thoroughly the biosynthesis pathways, we compared transcriptomics of different anther stages between *SSTL*-*F* and *sstl*-*s* (Fig. 6). The heat map illustrated five clusters with specific differentially expressed genes. Clusters I and II were upregulated genes from *sstl*-*s* in LpM and YM stages, clusters III and IV were specifically upregulated genes from *SSTL*-*F* in LpM or YM stages individually, and cluster V genes were upregulated in both YM and LpM stages from *SSTL*-*F* (Fig. 6a). From these five clusters, several genes listed in Additional file 5: Table S3 were selected for validation with real-time q-PCR analysis (Fig. 6b, c). The expression pattern between RNA-seq and q-PCR was similar and had almost equal log_2_ fold-change. The q-PCR expression of these selected genes also matched the heat map distribution in the five clusters; for example, genes in Os03g0236200, Os04g0486600, and Os10g0167300 were upregulated only in *sstl*-*s*; Os10g0524500, Os03g0429800, Os01g0854800 and Os08g0131100 of *SSTL*-*F* were significantly upregulated in YM with more than two-fold change on both RNA-seq and q-PCR; Os11g0210100, Os03g0234900, Os05g0209600, and Os05g0518300 of *SSTL*-*F* were particularly highly expressed in LpM; and Os02g0626100, Os02g0106100, Os05g0542800, and Os06g0156600 of *SSTL*-*F* had two-fold higher expression in both YM and LpM (Fig. 6b, c). Meanwhile, two late pollen preference genes, Os09g0381400 (similar to Ervatamin C) and Os09g0388400 (HAD-superfamily hydrolase-like), were also expressed in fertile pollen grains (Fig. 6b, c) (Moon et al. 2018). The expression of Os09g0388400 was 16-fold higher in fertile *SSTL*-*F* than *sstl*-*s*, which indicated that the sterile *sstl*-*s* anther contained a high amount of defective pollen grains.

The transcriptomics analysis also revealed that the sterile *sstl*-*s* anther genes contained low expression of biosynthesis pathway genes such as xanthine dehydrogenase (Os03g0429800), which participates in purine metabolism; the catalytic cycle of cytochrome P450 (Os01g0854800 and Os08g0131100), involved in secondary metabolites synthesis for exine formation; class III peroxidase (Os11g0210100 and Os03g0234900), which uses electrons in ox-reduction reaction to manipulate phenolic compound synthesis and secondary metabolites; GDSL esterase (Os05g0209600, Os05g0518300, and Os06g0156600), related to lipolytic enzymes and thus controlling pollen wall structure; fructosyl transferase (Os02g0106100), participating in starch composition of mature pollen grains; and the polygalacturonases-like 9 (Os05g0542800), involved in carbohydrate metabolism of polysaccharides for intine utilization. We used Rice Anther Expression Plots analysis to confirm that these significant transcripts of pollen development were expressed during anther-specific stages (Additional file 6: Figure S3). For instance, genes that appear from later meiosis to uninucleate microspore stage were consistent with our data in the YM stage. No Pollen 1 (Os10g0524500), xanthine dehydrogenase (Os03g0429800), and cytochrome P450 86A7-2 (Os01g0854800) were specifically upregulated in YM only, which was identical to the pattern in Rice Anther Expression Plots analysis. Os02g0106100, Os05g0542800, Os11g0210100, and Os09g0388400, significant genes in LpM stage, also responded to the same stage of bicellular pollen and mature pollen stage as in Rice Anther Expression Plots.

## Discussion

### Somaclonal variation might cause the *sstl* trait

On cosegregation of the T-DNA insertion and sterile phenotype of two TRIM mutants M0024091 and M0017896, SUMO E3 ligase *SIZ1* and *bHLH142* were found to control male sterility via anther dehiscence and PCD of tapetum (Thangasamy et al. 2011; Ko et al. 2014). However, in the current study, the phenotype (sterility) and genotype (T-DNA insertion) did not cosegregate in the TRIM mutant M0037841. Previous studies have shown that rice T-DNA insertional mutants, such as *Tos17* and TRIM, show low tagging efficiency (Droc et al. 2013; Wei et al. 2016b; Wu et al. 2017). The transformation was frequently used to study both forward and reverse genetics in knockdown/knockout mutants, overexpression lines, and T-DNA activation tagging lines, etc. In rice transformation, the procedure included repetitive tissue culture steps such as rice embryogenic callus generation, antibiotic selection of callus, and seedling regeneration. This long-term cullus growth period may lead to somaclonal variation including single nucleotide polymorphism (SNP), indels, chromosome doubling, and chromosome translocations (Wei et al. 2016a). From 600 to 2000 SNPs/indels per plant may occur during rice regeneration and transformation in the TRIM mutants (Wei et al. 2016a). Thus, this M0037841 semi-sterile *sstl* mutation might be also caused by somaclonal variations during transformation.

From the developmental morphology of *sstl* progeny segregates, the completely sterile *sstl*-*s* line inherited the sterility and showed defects in anther locule and pollen grain. This kind of somaclonal variation might be applied to crop improvement with stable heritability and also may speed up the breeding process and replace traditional crossbreeding (Jain 2001). Additional file 2: Table S2 illustrates that 6 T-DNA insertional mutant lines have normal fertility. From TRIM database information, we already know that the T-DNA insertion site of M0037841 locates in the exon of a *Mei2*-*like* protein (Os02g0517531). *Mei2*-*like* of *Arabidopsis* is a regulator of meiosis and expressed in gametes of pollens (Kaur et al. 2006). The rice genome contains 6 *OsMei2*-*like* genes (Anderson et al. 2004). Thus, M0037841 might maintain fertility because of the gene abundance.

### Defective events start in microsporangia in the *sstl*-*s* mutant

From the cytological observation of anther morphology, we investigated the microspores of the sterile mutant *sstl*-*s* from YM to Mp stages and discovered that the early stage of microspore development is unusual and defective (Fig. 3f–o). The expression of the later pollen preference gene HAD-superfamily hydrolase-like (Os09g0388400) is extremely high in fertile *SSTL*-*F*, which indicates a severe defect in this expression in the mutant *sstl*-*s*. Significant downregulation of HAD-superfamily hydrolase-like may occur because *sstl*-*s* has empty defective pollen or even no microspores produced in anther locules (Fig. 3f–o). Moreover, the mutant *sstl*-*s* has abnormal and defective anthers in slender twig-like or even “X”-like shapes during microsporangia development stage (Additional file 4: Figure S2). A recent study of the rice SQUAMOSA PROMOTER-BINDING PROTEIN-LIKE 6 (SPL6) mutant *spl6*-*1* also showed a cell death phenotype in the panicle and the distorted anther in a “X”-like shape, signaling damaged microsporangia development (Wang et al. 2018). *OsSPL6* directly repressed inositol-requiring enzyme 1 (*IRE1*) expression as a transcriptional repressor. Also, overexpressed *IRE1* was an endoplasmic reticulum stress sensor resulting in a cell death phenotype of panicle (Wang et al. 2018). Un-developmental microsporangia of the *spl6*-*1* mutant led to sterility, including white spikelet and yield loss; the mutant also showed a significant reduction in panicle length, spikelet number, seed setting rate, and 1000 grain-weight. However, the *spl6*-*1* mutant had no effect on plant height and number of panicle branches and thus was similar to *sstl*-*s*. In *Arabidopsis*, the expression of *SPL1* and *SPL12* increased heat tolerance during the reproductive stage and promoted seed production under heat stress. Without functional *SPL1* and *SPL12*, the inflorescences of *Arabidopsis* showed dead cells and seeds were aborted (Chao et al. 2017). From morphological characterization, the microsporangia development of *sstl*-*s* has been destroyed, similar to the anthers of the *spl6*-*1* mutant with an “X”-shape of early developmental anthers. From the genome sequence data, we confirm that *sstl*-*s* has no mutation in *OsSPL6* (data not shown). The defective pollen and the “X”-like defective anther of *sstl*-*s* imply that microsporangia development is defective; thus, no sporogenous cells are produced in abnormal anther locules during early anther development.

### Several biosynthetic pathways are affected in *sstl*-*s*

**Fig. 7:**
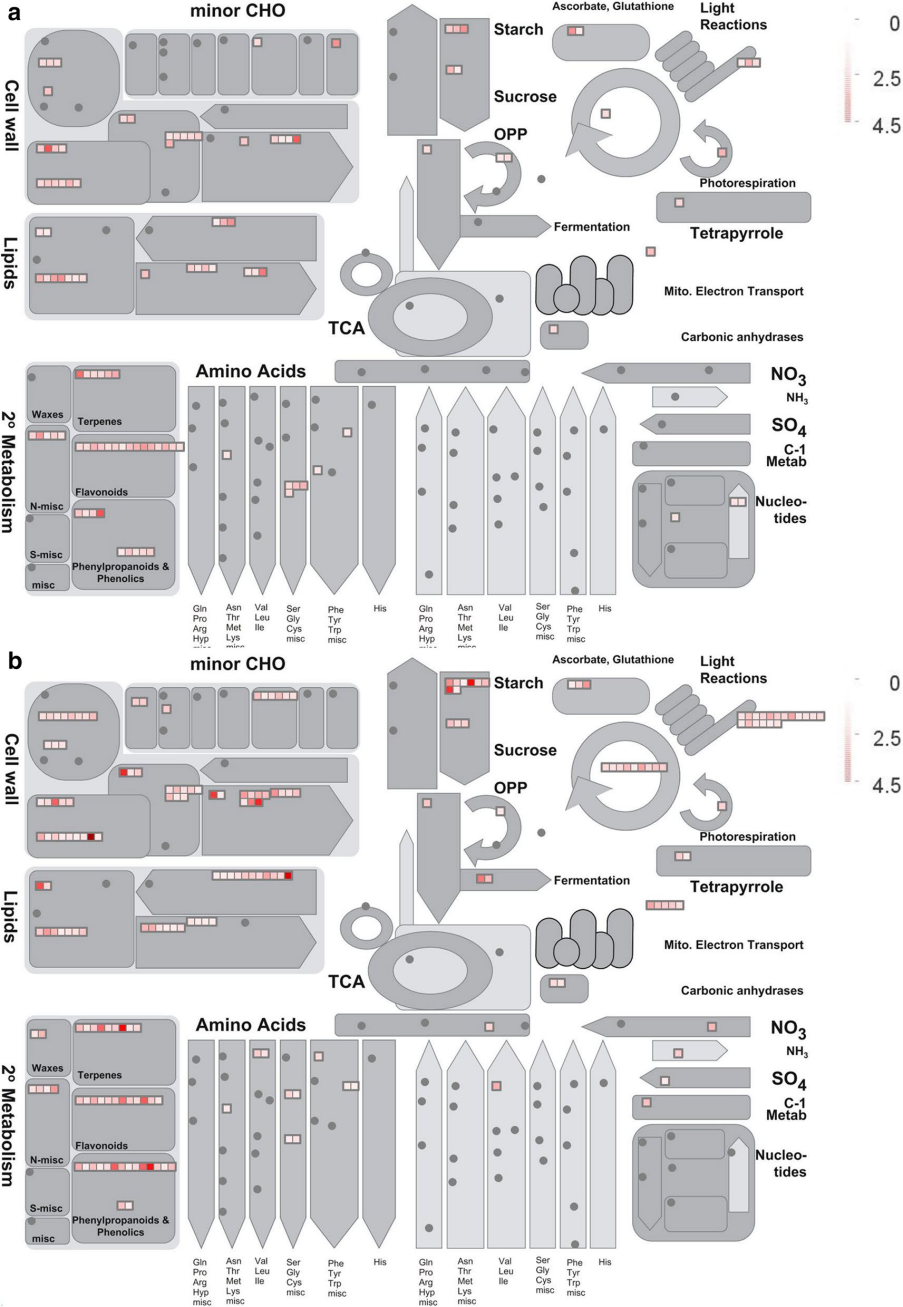
MapMan analysis of fertile upregulation genes in different anther stages. Metabolism overview of MapMan analysis in fertile upregulated genes at **a** YM stage and **b** LpM stage

Our studies indicate the pollen in the mutant *sstl*-*s* is severely reduced, with abnormal pollen wall in exine and intine, shrunken pollen grains, low pollen viability and no pollen grain germination. From comparative transcription analysis, the RNA-seq data was substantiated by q-PCR assay illustrating that our silicon data are as reliable as the actual expression on real-time q-PCR (Fig. 6b, c). We evaluated the difference in transcriptomics between *sstl*-*s* and *SSTL*-*F* as compared with reference homologs from the KEGG database and tried to understand the multiple impacts on sterility. From the 5 significant transcript heat map clusters (Fig. 6a) and EXPath Tool analysis (Additional file 7: Table S4), clusters I and II revealed that transcripts of *sstl*-*s* are involved in degradation of carbon metabolism and amino acid metabolism, such as glyceraldehyde-3-phosphate dehydrogenase (GAPDH) (Os04g0486600), enolase 2 gene (Os10g0167300), and glutamate decarboxylase 3 (*OsGAD3*) (Os03g0236200). In gluconeogenesis, GAPDH participates in glycolysis at the 6th step to break down glucose for energy contribution and enolase 2 catalyzes 2-phosphoglycerate (2-PG) to phosphoenolpyruvate from the 9th or second to the last step of glycolysis (Yang et al. 2015; Swain et al. 2017). The expression of *OsGAD3*, which catalyzes glutamate to γ-aminobutyric acid for glutamate degradation (Zhang et al. 2007; El-Kereamy et al. 2012), was increased in *sstl*-*s*. In clusters III, IV, and V, the upregulated genes of fertile *SSTL*-*F* share similar functions, such as phenylalanine metabolism, cutin/suberine/wax biosynthesis, secondary metabolites, flavone/flavonol biosynthesis, and starch/sucrose metabolism (Additional file 7: Table S4). We also performed MapMen analysis and illustrated that biosynthetic pathways in cell wall, lipids, secondary metabolism, and starch are affected in *sstl*-*s* (Fig. 7). Integral pollen exine was controlled by genes related to fatty acid components, secondary products, and cutin/wax biosynthesis for pollen wall conformation (Quilichini et al. 2015; Shi et al. 2015). Therefore, metabolic events are important in sporophytic development. For instance, a well-developed exine of pollen has a tightly bound fatty acid-like sporopollenin that organizes a bridge-like structure and then the fatty acid assembles aliphatic derivatives such as cutin and wax to form the pollen wall and anther cuticle (Li et al. 2010; Li-Beisson et al. 2013). These functional metabolisms in lipids and fatty acid are consistent with the observation of a rich lipid structure in *SSTL*-*F* exine TEM ultrathin section characterizations (Fig. 4). Meanwhile, lipid participates in phenolic secondary metabolites in flavonoid production. The flavonoids function as antioxidants to protect the plant against abiotic stress and biotic stress to clean superoxide anions O_2_^−^, hydrogen peroxide (H_2_O_2_), hydroxyl radicals (OH^−^), and singlet oxygen (^1^O_2_), etc. when these oxygen species (ROS) accumulate under stress (Hernández et al. 2009). In rice flowering stage, flavonoids protect anthers and pollens against ultraviolet damage and increase the bright yellow color to attract pollinators (Thompson et al. 2010; Ning et al. 2018). Our data illustrate that sterile *sstl*-*s* shows downregulated flavonoid synthesis-related genes (Fig. 7 and Additional file 7: Table S4). Figure 2b, c also show white anthers and pollen grains, indicating no flavonoid accumulation. A male-sterile maize mutant of irregular pollen exine 1 (*ipe1*) also had defective pollen grains and abnormal pollen exine with low wax and flavonoid accumulation (Chen et al. 2017). With the secondary metabolomics essential for pollen development, any defect of these controlling genes may cause sterility and loss of yield production. The oxidative phosphorylation pathway may regulate secondary metabolites production and *Arabidopsis* soluble pyrophosphatases (*PPa3*) specifically present in root hairs and pollen tissues (Gutiérrez-Luna et al. 2018; Segami et al. 2018). Our transcriptional data also demonstrate that pyrophosphatase-related genes are upregulated only in fertile anthers and they might participate in a secondary metabolic pathway for pollen wall structure. The starch accumulation of mature pollen is initiated from glucose-6-phosphate (Glc-6-P), which converts to Glc-1-P, and Glc-1-P then converts to ADP-glucose in the amyloplasts that depend on plastidic ADP-Glc synthesis (Lee et al. 2016). The starch in pollen grains may offer energy for pollen grains in maturation and germination. The I_2_KI staining (Fig. 2k) of wild-type and mutant pollen agrees with the expression difference in starch synthesis genes (Fig. 7 and Additional file 7: Table S4).

We also investigated several genes related to male-sterile phenotype. Rice No Pollen 1 (*NP1*) (Os10g0524500) of fertile pollen is expressed up to threefold that of the sterile *sstl*-*s* (Fig. 6b and Additional file 5: Table S3). *NP1* regulates anther size and Ubisch body formation to contribute a pollen exine structure and manipulate fertility in pollen grains (Liu et al. 2017). The *np1*-*4* mutant has an irregular pollen granule, reductive cutin, and less cuticular wax in exine formation (Liu et al. 2017). Rice lipid hydrolysis-related genes GDSLs are upregulated in mature pollen of *SSTL*-*F* (Fig. 6b–c and Additional file 5: Table S3). One GDSL was confirmed from *Brassica rapa*, known as extracellular lipase 6 (*BrEXL6*), which functions as a pollen development-related gene. Another case was *ZmMs30*, a maize GDSL, which was found essential for male fertility because the knockout *ZmMs30* mutant showed a disordered pollen wall and defective pollen grains (Ji et al. 2017; An et al. 2019).

## Conclusion

Here we report an untagged T-DNA insertional mutant M0037841 that has defective pollen and abnormal anthers from its *sstl* progeny segregaters. The abnormal anther locule is related to the damaged microsporangia and results in no microspore generation. The defective anther locule of *sstl*-*s* also illustrates the importance of the anther tapetum, which provides lipid-like sporopollenin for pollen maturation. Transcripts of sterile *sstl*-*s* are downregulated in several secondary-metabolism biosynthesis pathways and lead to irregular exine and absent intine in *sstl*-*s*. This information reveals the defective pollen grains of *sstl*-*s* and coincides with the irregular pollen wall and loss of pollen viability. Comparative transcriptomics analysis illustrates that the mechanism of male sterility is related to biosynthetic pathways for cell wall, lipids, secondary metabolism, and starch accumulation. We illustrate the importance of microsporangia development and male fertility of microspores. A series of morphological observations of mutant *sstl*-*s* anther demonstrated that the defective pollen grains are caused by unproduced intine and unfunctional exine of the pollen wall, then the defective pollen of *sstl*-*s* do not germinate and become sterile.

## Additional files

**Additional file 1: Table S1.** Primers list.

**Additional file 2: Table S2.** Genotyping and phenotyping analysis of M0037841 segregants.

**Additional file 3: Figure S1.** Panicle development of *sstl* progeny segregates. **a, c, f** *SSTL-F* plants show the fertile panicles. **b, d, e** *sstl-s* mutant shows sterile spikelets during reproductive stage.

**Additional file 4: Figure S2.** Structure analysis of the undeveloped anther *sstl-s*. **a-c** Cross section and **d-i** TEM analysis showing abnormal anthers of *sstl-s* in the early stage. **(a-c)** Bar=40 μm. **(d-f)** Bar=2 μm. **(g-i)** Bar=1 μm.

**Additional file 5: Table S3.** Genes selected from RNA-seq heat-map analysis.

**Additional file 6: Figure S3.** The differential expressed genes of *SSTL-F* and *sstl-s* during anther developing stages using Rice Anther Expression Plots (https://www.cpib.ac.uk/anther/riceindex.html). PM=Premeiosis; M=Meiosis; UM=Uninucleate Microspore; BP=Bicellular Pollen; TP=Tricellular pollen; MP=Mature Pollen.

**Additional file 7: Table S4.** Prediction of gene function in RNA-seq heat-map analysis result.

## Data Availability

Not applicable.

## Abbreviations

bHLH: basic helix-loop-helix
EAT1: ETERNAL TAPETUM1
EMS1: EXCESS MICROSPOROCYTES1
EpM: early pollen mitosis stage
GDSL: Gly-Asp-Ser-Leu
GM filed: genetically modified plants isolation field
I_2_–KI: iodine–potassium iodide
LpM: late pollen mitosis stage
Mp: mature pollen stage
NP1: Rice No Pollen 1
PCD: programmed cell death
PMC: pollen mother cells
PPC: primary parietal cells
PS: primary sporogenous
SEM: scanning electron microscopy
SNP: single nucleotide polymorphism
SPC: secondary parietal layers
SPL: SQUAMOSA PROMOTER-BINDING PROTEIN-LIKE
sstl: semi-sterile mutant
SSTL-F: fertile plants of sstl progeny segregates
sstl-s: sterile plants of sstl progeny segregates
sstl-ss: semi-sterile plants of sstl progeny segregates
TARI: Taiwan Agricultural Research Institute
TDR: TAPETUM DEGENERATION RETARDATION
TEM: transmission electron microscope
TPD1: TAPETUM DETERMINANT1
TRIM: Taiwan Rice Insertional Mutagenesis
VP: vacuolated pollen stage
YM: young microspore stage
