# Supplementary material for: Studies of a rice sterile mutant sstl from the TRIM collection
Source: Bot Stud. 2019 Jul 10;60:12. doi: 10.1186/s40529-019-0260-3 (PMC6620220; doi:10.1186/s40529-019-0260-3)
Supplement: Supplementary file 2 — Additional file 2: Table S2. Genotyping and phenotyping analysis of M0037841 segregants. [file 40529_2019_260_MOESM2_ESM.docx]

**Table S2** **genotyping and phenotyping analysis of M0037841 segregants**

| **No.** | **NP** | **GWP**  **(g)** | **P-type** | **G-type *GUS*** | **G-type**  ***HPT*** | **G- type**  ***35SE*** | **No.** | **NP** | **GWP**  **(g)** | **P-type** | **G-type *GUS*** | **G-type**  ***HPT*** | **G-type**  ***35SE*** |
| --- | --- | --- | --- | --- | --- | --- | --- | --- | --- | --- | --- | --- | --- |
| 1 | 15 | 0.81 | sterile | W | W | W | 14 | 15 | 3.68 | fertile | W | W | W |
| 2 | 13 | 0.75 | sterile | NA | NA | NA | 15 | 12 | 2.99 | fertile | W | W | W |
| 3 | 24 | 0.47 | sterile | W | W | W | 16 | 11 | 2.57 | fertile | W | W | W |
| 4 | 20 | 0.55 | sterile | W | W | W | 17 | 16 | 3.09 | fertile | W | W | W |
| 5 | 12 | 3.45 | fertile | W | T | T | 18 | 6 | 0.58 | sterile | W | W | W |
| 6 | 8 | 2.44 | semi-s | W | W | W | 19 | 13 | 0.92 | sterile | W | T | T |
| 7 | 10 | 0.75 | sterile | W | T | T | 20 | 18 | 3.7 | fertile | W | T | T |
| 8 | 15 | 0.61 | sterile | W | W | W | 21 | 17 | 2.16 | semi-s | W | W | W |
| 9 | 11 | 3 | fertile | W | T | T | 22 | 13 | 3.55 | fertile | W | T | T |
| 10 | 13 | 0.69 | sterile | W | W | W | 23 | 8 | 3.84 | fertile | W | W | W |
| 11 | 14 | 2.75 | fertile | W | W | W | 24 | 30 | 0.68 | sterile | W | W | W |
| 12 | 10 | 3.15 | fertile | W | T | T | 25 | 17 | 0.7 | sterile | W | T | T |
| 13 | 17 | 0.76 | sterile | W | T | T | 26 | 28 | 3.19 | fertile | W | T | T |

NP=number of panicles per plant; GWP=grain weight per panicle; P-type=phenotype of the plant; G-type=genotype of plant; *GUS*= *GUS* gene, *HPT*=hygromycin phosphotransferase gene; *35SE*=*CaMV35S* enhancer; semi-s=semi-sterile; W=genotype same as wild-type background without T-DNA insertion; T=with T-DNA insertion; NA= unavailable data due to the low amount of genomic DNA.
