# Supplementary material for: Studies of a rice sterile mutant sstl from the TRIM collection
Source: Bot Stud. 2019 Jul 10;60:12. doi: 10.1186/s40529-019-0260-3 (PMC6620220; doi:10.1186/s40529-019-0260-3)
Supplement: Supplementary file 3 — Additional file 3: Figure S1. Panicle development of sstl progeny segregates. a, c, f SSTL-F plants show the fertile panicles. b, d, e sstl-s mutant shows sterile spikelets during reproductive stage. [file 40529_2019_260_MOESM3_ESM.pdf]

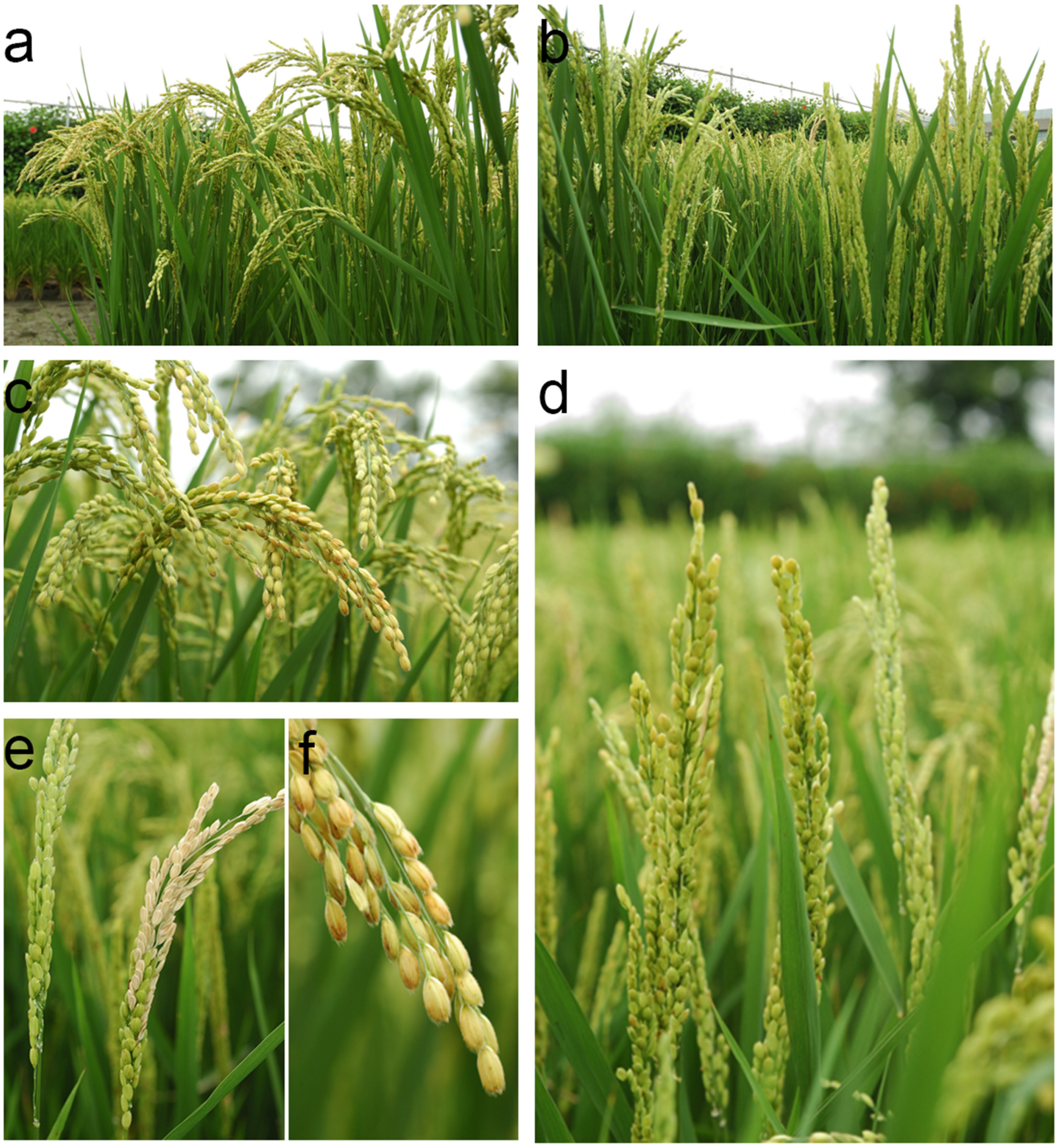

Fig. S1 Panicle development of *sstl* progeny segregates. **a, c, f** *SSTL-F* plants show the fertile panicles. **b, d, e** *sstl-s* mutant shows sterile spikelets during reproductive stage.
