# Supplementary material for: Studies of a rice sterile mutant sstl from the TRIM collection
Source: Bot Stud. 2019 Jul 10;60:12. doi: 10.1186/s40529-019-0260-3 (PMC6620220; doi:10.1186/s40529-019-0260-3)
Supplement: Supplementary file 4 — Additional file 4: Figure S2. Structure analysis of the undeveloped anther sstl-s. a-c Cross section and d-i TEM analysis showing abnormal anthers of sstl-s in the early stage. (a-c) Bar=40 μm. (d-f) Bar=2 μm. (g-i) Bar=1 μm. [file 40529_2019_260_MOESM4_ESM.pdf]

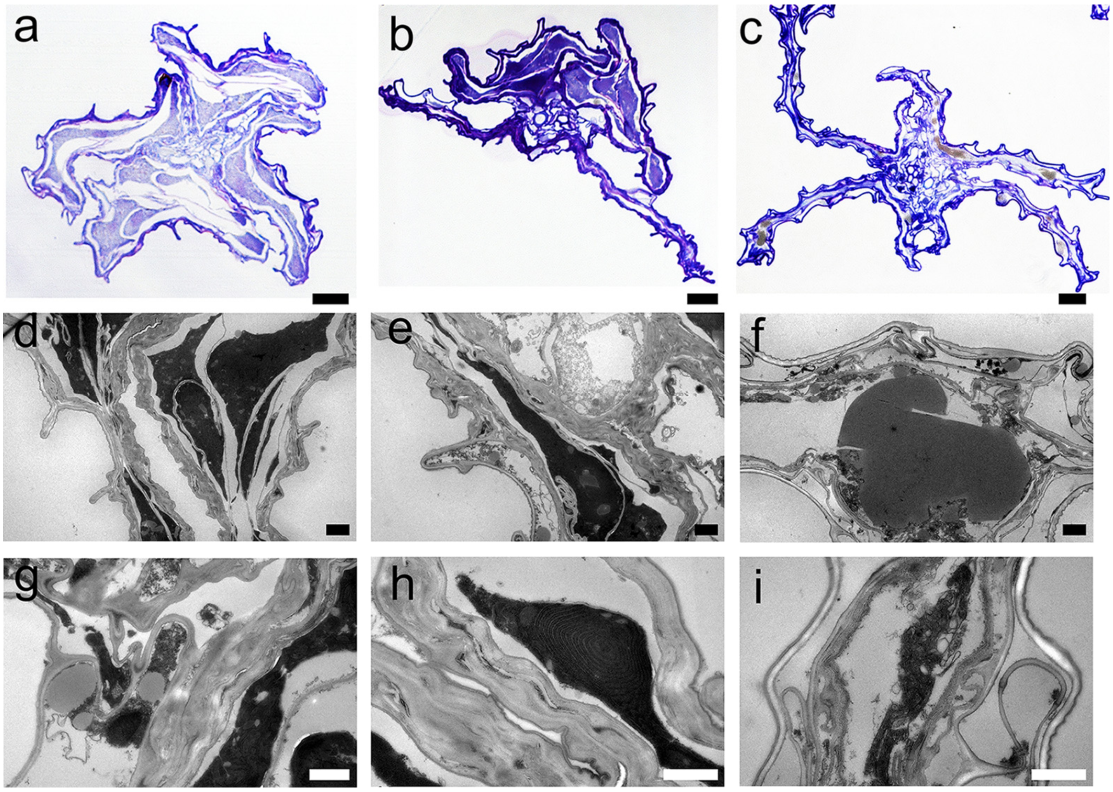

Fig. S2 Structure analysis of the undeveloped anther *sstl-s*. **a-c** Cross section and **d-i** TEM analysis showing abnormal anthers of *sstl-s* in the early stage. **(a-c)** Bar=40 μm. **(d-f)** Bar=2 μm. **(g-i)** Bar=1 μm.
