# Supplementary material for: Studies of a rice sterile mutant sstl from the TRIM collection
Source: Bot Stud. 2019 Jul 10;60:12. doi: 10.1186/s40529-019-0260-3 (PMC6620220; doi:10.1186/s40529-019-0260-3)
Supplement: Supplementary file 5 — Additional file 5: Table S3. Genes selected from RNA-seq heat-map analysis. [file 40529_2019_260_MOESM5_ESM.docx]

**Table S3 Genes selected from RNA-seq heat-map analysis**

| RAP-DB ID. | Description/Gene Name Synonym | YM^(a)^ F/s | LpM^(b)^ F/s |
| --- | --- | --- | --- |
| Os03g0236200 | glutamate decarboxylase 3 | -1.65 | -0.62 |
| Os04g0486600 | Glyceraldehyde-3-phosphate dehydrogenase | -0.38 | -0.61 |
| Os10g0167300 | Similar to Enolase 2 | -0.14 | -0.54 |
| Os10g0524500 | No Pollen 1 | 1.61 | 0.44 |
| Os03g0429800 | Xanthine dehydrogenase | 1.23 | 0.16 |
| Os07g0513000 | ATP synthase g-chain | 0.28 | 1.00 |
| Os01g0854800 | Cytochrome P450 86A7-2 | 2.00 | 0.65 |
| Os08g0131100 | cytochrome P450 hydroxylase 703A3 | 1.07 | -0.02 |
| Os02g0626100 | phenylalanine ammonia lyase 1 | 0.93 | 1.00 |
| Os08g0498100 | caffeoyl-CoA O-methyltransferase | 0.80 | 0.57 |
| Os02g0735200 | cytosolic glutamine synthetase 1 | 0.35 | 0.71 |
| Os02g0106100 | fructosyl transferase, vacuolar invertase 2 | 0.94 | 1.83 |
| Os05g0542800 | Polygalacturonases-Like 9 | 1.07 | 1.68 |
| Os07g0208500 | Cellulose synthase A catalytic subunit 8 | 0.63 | 0.98 |
| Os11g0210100 | class III peroxidase 133 | 1.09 | 1.99 |
| Os03g0234900 | class III peroxidase 39 | 0.82 | 1.76 |
| Os05g0209600 | GDSL esterase/lipase protein 63 | 0.19 | 1.42 |
| Os05g0518300 | GDSL esterase/lipase protein 73 | 0.50 | 1.82 |
| Os06g0156600 | GDSL esterase/lipase protein 77 | 0.92 | 1.30 |
| Os09g0381400 | Similar to Ervatamin C/cysteine protease | 0.87 | 1.71 |
| Os09g0388400 | HAD-superfamily hydrolase-like | 3.87 | 3.50 |

(a) RNA-seq *SSTL-F*/*sstl-s* log_2_ fold-changes in young microspore (YM) stage.

(b) RNA-seq *SSTL-F*/*sstl-s* log_2_ fold-changes in late pollen mitosis (LpM) stage.
