# Supplementary material for: Studies of a rice sterile mutant sstl from the TRIM collection
Source: Bot Stud. 2019 Jul 10;60:12. doi: 10.1186/s40529-019-0260-3 (PMC6620220; doi:10.1186/s40529-019-0260-3)
Supplement: Supplementary file 6 — Additional file 6: Figure S3. The differential expressed genes of SSTL-F and sstl-s during anther developing stages using Rice Anther Expression Plots (https://www.cpib.ac.uk/anther/riceindex.html). PM=Premeiosis; M=Meiosis; UM=Uninucleate Microspore; BP=Bicellular Pollen; TP=Tricellular pollen; MP=Mature Pollen. [file 40529_2019_260_MOESM6_ESM.pdf]

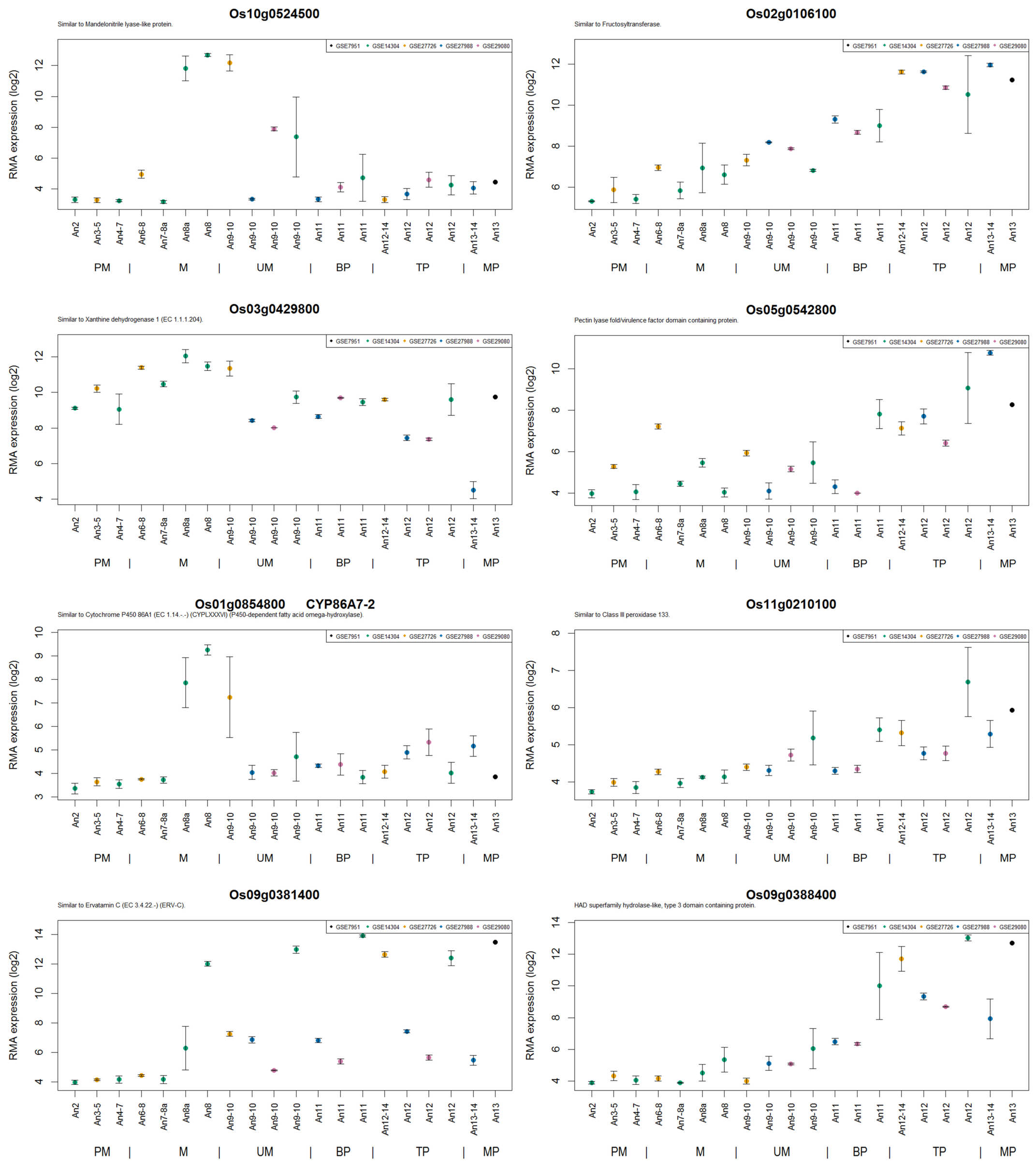

Fig. S3 The differential expressed genes of *SSTL-F* and *sstl-s* during anther developing stages using Rice Anther Expression Plots (https://www.cpib.ac.uk/anther/riceindex.html). PM=Premeiosis; M=Meiosis; UM=Uninucleate Microspore; BP=Bicellular Pollen; TP=Tricellular pollen; MP=Mature Pollen.
